# Board Exam Pass Rates: Impacted by Residency Length or Residency Strength?

**DOI:** 10.1002/aet2.70213

**Published:** 2026-06-11

**Authors:** David Wilson, Pauline Wiltz, Alexandra Sappington, Lauren Diercks

**Affiliations:** ^1^ Department of Emergency Medicine University of Cincinnati Cincinnati Ohio USA; ^2^ Elson S. Floyd College of Medicine Washington State University Richland Washington USA; ^3^ Department of Emergency Medicine Louisiana State University New Orleans New Orleans Louisiana USA; ^4^ Department of Emergency Medicine Stanford University Palo Alto California USA

To the Editor,

As the Emergency Medicine community awaits an update from the Accreditation Council for Graduate Medical Education (ACGME) on residency program requirements, the study by Topping et al. published in *AEM E&T* on American Board of Emergency Medicine (ABEM) Qualifying Examination (QE) pass rates adds to the conversation [1]. While the conclusions of other similar studies have some disagreement, one finding is increasingly difficult to ignore: the age of a residency training program, rather than the length of training, appears to have a real impact on QE success [2, 3].

The conversation surrounding a universal transition to 4‐year Emergency Medicine residency programs has been framed around improving educational outcomes and ensuring resident preparedness [4]. However, these data suggest that ABEM QE pass rates are not substantially different between residents who graduate from 3‐ and 4‐year programs. Instead, a more striking signal appears tied to the age and maturity of the residency program itself.

Established programs benefit from years of curricular refinement, experienced faculty development, institutional support, alumni feedback, and stable educational infrastructure that newer programs lack. As Topping et al. suggest, there are additional program‐level factors for researchers to investigate, such as Council of Teaching Hospital designation and hospital ownership, both of which likely relate to institutional support for the educational environment. If ABEM QE pass rates differ primarily by program maturity and educational infrastructure rather than by training length, a sweeping mandate extending all programs to 4 years risks addressing the wrong problem at a high cost.

Residency standards should be strengthened, and the Emergency Medicine community is anxiously awaiting final changes from the ACGME. Higher expectations for patient volume, procedural experience, faculty engagement, scholarly activity, and leadership in critical resuscitations have broad community support for improving program quality. A struggling educational environment does not become stronger by adding twelve more months. Otherwise, efforts should focus on improving the rigor of program certification, ensuring adequate resources for newer programs during their formative years, and strengthening accountability for underperforming residencies.

As the future of emergency medicine training is under review, evidence cautions not to mistake the duration of training for its quality. The ABEM QE data highlights the importance of mature educational infrastructure rather than a need for universally longer training. Mandating a four‐year curriculum risks an overly broad solution to a narrower problem. In order to produce better prepared emergency physicians, the focus should be on strengthening program quality, support for developing residencies, and accountability. The best interventions in improving the practice of emergency medicine are targeted, evidence‐based, and directed at the true source of the problem. Residency reform should be held to the same standard.

## Author Contributions

**David Wilson:** conceptualization, writing – review and editing, writing – original draft. **Pauline Wiltz:** conceptualization, writing – original draft, writing – review and editing. **Lauren Diercks:** writing – review and editing, conceptualization. **Alexandra Sappington:** writing – review and editing.

## Funding

The authors have nothing to report.

## Conflicts of Interest

D.W., P.W. and A.S. serve on the Emergency Medicine Residents Association Board of Directors. D.W., P.W. and A.S. receive no financial compensation for their service. L.D. serves on the Society of Academic Emergency Medicine Resident and Medical Students Section Board. L.D. receives no financial compensation for their service. The authors declare no conflicts of interest.

## Data Availability

Data sharing not applicable to this article as no datasets were generated or analysed during the current study.

## References

[1] C. E. W. Topping , A. K. Venkatesh , C. Rothenberg , et al., “Board Examination Pass Rates of Emergency Medicine Residency Training Programs: Associations With Founding Year, Teaching Status, Length, and Hospital Ownership,” AEM Education and Training 10, no. 2 (2026): e70144, 10.1002/aet2.70144. 41798586 PMC12964488

[2] S. R. White , M. Gottlieb , F. K. Ankel , et al., “Declining Performance on the Qualifying Examination: Modeling of a Potential Inflection Point in Emergency Medicine,” Journal of the American College of Emergency Physicians Open 7, no. 2 (2026): 100333, 10.1016/j.acepjo.2026.100333. 41726089 PMC12924182

[3] P. Nasrallah , Associations Between American Board of Emergency Medicine Examination Pass Rates and Residency Program Characteristics and Hospital/Physician Group Ownership Type, 2021–2023 (Abstract presented at: Society for Academic Emergency Medicine Academic Assembly, 2026).

[4] ACGME EM RC Writing Group , Proposed Emergency Medicine Program Requirements (Accreditation Council for Graduate Medical Education, 2025), https://www.acgme.org/globalassets/pfassets/reviewandcomment/2025/110_emergencymedicine_rc_02122025.pdf.

